# Telemedicine for headache disorders in real-world practice: from patient experience to health system impact

**DOI:** 10.1007/s10072-026-09205-y

**Published:** 2026-07-04

**Authors:** Roberta Grasso, Elena Carapelle, Giuseppe Raunich, Carlo Avolio

**Affiliations:** 1 Department of Neurology, Azienda Ospedaliero-Universitaria “Ospedali Riuniti” di Foggia, Foggia, Italy; 2 Public sector employee, Potenza, Italy; 3 Department of Neurology, University of Foggia, Foggia, Italy

**Keywords:** Telemedicine, Headache disorders, Patient satisfaction, Real word practice

## Abstract

**Background:**

Telemedicine has expanded rapidly in neurological care and is increasingly applied to headache management to improve access and continuity of follow-up. However, patient experience and healthcare system impact in real-world practice remain insufficiently explored.

**Objective:**

The objective of this narrative review is to evaluate clinical effectiveness, patient experience, and healthcare system impact in the management of headache disorders.

**Methods:**

A narrative review of the literature was conducted, including randomized controlled trials, observational studies, real-world evidence contributions, and reviews addressing telemedicine in headache care. Evidence was synthesized thematically, focusing on clinical outcomes, patient acceptance, and implementation aspects.

**Results:**

Available evidence supports the clinical effectiveness and safety of telemedicine in selected headache patients, particularly in non-acute settings and structured follow-up pathways. Outcomes assessed using validated instruments (HIT-6, MIDAS, VAS) are generally comparable to in-person visits. High patient satisfaction and willingness to continue telemedicine are consistently reported, mainly due to convenience and time and cost savings. Real-world studies suggest telemedicine may improve access to specialist care and optimize healthcare resource utilization, although challenges related to digital literacy, organizational requirements, and equity remain.

**Conclusions:**

Telemedicine is a valuable component of contemporary headache management when integrated into structured clinical pathways. Beyond clinical effectiveness, patient experience and organizational context are critical for successful implementation. Future research should focus on standardized care models, larger populations, and long-term real-world outcomes.

## Introduction

In recent years, telemedicine has undergone rapid evolution also in the field of neurological diseases, fostering new models of healthcare delivery and alternative modes of interaction between physicians and patients. Its application appears particularly promising in the management of chronic conditions, which require continuous monitoring and frequent follow-up visits [1].

Despite the growing interest in and diffusion of telemedicine tools, the available evidence remains heterogeneous and partly limited. In particular, few studies have systematically explored the feasibility and acceptance of these tools from the patient perspective key aspects for the effective integration of telemedicine into routine clinical practice [2].

In the context of headache disorders, which are highly prevalent and have a significant impact on quality of life [3], telemedicine represents a potentially relevant opportunity to improve access to care and continuity of treatment. In addition to improving access to care, this telemedicine-based approach has the potential to optimize healthcare resource utilization by reducing unnecessary in-person visits, minimizing travel-related burdens for patients, and decreasing time constraints for both patients and healthcare providers. These aspects are particularly relevant in chronic conditions such as headache disorders, where frequent follow-up is required and healthcare systems often face limitations in specialist availability.

Furthermore, telemedicine may facilitate the delivery of a broader range of therapeutic options for patients with headache, including pharmacological management, behavioral interventions, patient education, and structured follow-up programs. This flexible approach may enhance treatment, personalization and continuity of care, particularly in patients requiring long-term management.

However, the role of telemedicine in headache management has not yet been fully clarified, especially with regard to patient experience and its impact on real-world clinical practice [4, 5].

Previous evidence has partially explored digital health approaches in headache management. In particular, a systematic review [6] focused on electronically delivered behavioral interventions, highlighting their feasibility while also emphasizing the limited and heterogeneous nature of the available evidence. However, this earlier work primarily addressed specific behavioral modalities rather than the broader implementation of telemedicine in clinical practice. Since then, the field has evolved rapidly, particularly following the COVID-19 pandemic [7], with a substantial increase in real-world applications of telemedicine across different stages of headache care. However, current evidence remains fragmented, often focusing on specific aspects such as clinical outcomes or digital interventions, without providing an integrated overview of patient experience and healthcare system impact in real-world settings. Therefore, an updated and more comprehensive synthesis focusing on patient experience and healthcare system impact is warranted. While several reviews and position statements have addressed telemedicine in headache care, most have primarily focused on specific aspects such as digital behavioral interventions, feasibility, or clinical outcomes. In contrast, the present narrative review aims to provide an integrated and updated perspective by simultaneously examining clinical effectiveness, patient experience, and health system impact within real-world practice. This combined focus allows for a more comprehensive understanding of how telemedicine can be effectively implemented across different stages of headache care, moving beyond isolated evaluations of efficacy toward a broader, practice-oriented framework. In light of these considerations, this narrative review aims to critically analyse the available literature on telemedicine in headache care, with a specific focus on its added value in real-world clinical practice, integrating evidence on clinical outcomes, patient experience, and healthcare system impact.

## Methods

This narrative review was conducted to synthesize the available evidence on telemedicine applications in headache management, with particular focus on clinical effectiveness, patient experience, and healthcare system impact.

A literature search was performed in PubMed/MEDLINE up to January 2026. Study selection was performed by screening titles and abstracts, followed by full-text evaluation of potentially relevant articles. The literature search yielded approximately 120 records. After removal of duplicates, about 95 articles were screened based on titles and abstracts. Of these, 50 studies were selected for full-text evaluation, and a final set of 30 articles was included in the qualitative synthesis. Although this review follows a narrative approach, the study selection process was guided by predefined eligibility criteria to enhance transparency.

The following keywords and their combinations were used: “telemedicine”, “telehealth”, “eConsult”, “remote consultation”, “headache”, “migraine”, “neurology”, “patient satisfaction”, “healthcare utilization”, and “real-world practice”.

Studies were considered eligible if they:

- Evaluated telemedicine interventions in patients with primary or non-acute headache disorders.
- Reported data on clinical outcomes, patient satisfaction, feasibility, safety, or healthcare utilization.
- Were randomized controlled trials, observational studies, cohort studies, pilot studies, or relevant narrative or systematic reviews.

The included studies were qualitatively synthesized according to thematic areas, including clinical effectiveness, patient experience, and healthcare system impact.

Articles focusing exclusively on acute emergency settings without specific headache data, pediatric populations without stratified analysis, or purely technical reports without clinical outcomes were excluded.

The final selection of studies was based on relevance to the objectives of this review. Given the narrative design, no formal quality scoring system was applied; however, particular attention was paid to study design, population characteristics, and outcome measures when interpreting the findings.

## Clinical effectiveness and safety of telemedicine in headache management

Clinical effectiveness and safety represent fundamental prerequisites for the adoption of telemedicine in headache management. To date, most of the available evidence has focused on non-acute headache conditions and on structured follow-up settings, in which telemedicine is used as an alternative to in-person visits rather than for initial diagnostic evaluation [8–10]. However, several limitations should be considered when interpreting these findings. Most studies have been conducted in selected populations and specialized settings, which may limit the generalizability of the results. Moreover, the available evidence primarily focuses on non-acute headache conditions, while data on the use of telemedicine in acute or emergency settings remain scarce. Further research is needed to better define its role across different clinical scenarios, particularly in acute headache management.

To provide a structured overview of the main available evidence, Table 1 summarizes primary studies, while Table 2 reports secondary literature (reviews, guidelines, and position statements) on telemedicine in headache management.

**Table 1:** Characteristics of the primary studies included in this narrative review evaluating telemedicine interventions for headache disorders, including study design, population, sample size, country/setting, telemedicine modality, comparator, follow-up, outcome measures, and main findings

Author, Year	Study Design	Population	Sample size	Country/Setting	Telemedicine modality	Comparator	Follow-up duration	Outcomes	Key Findings
Liu et al. [11]	Randomized controlled study	Migraine	147	China, tertiary headache center	Social software, telephone, e-mail. Short message	In person outpatient visits	3–6 months	Headache frequency, VAS, HIT-6, MIDAS, patient satisfaction, treatment adherence	Comparable efficacy, higher satisfaction vs. in-person
Chiang et al. [12]	Survey study	Headache disorder	1172	USA, multicenter (online survey)	Telemedicine visits	NR	NR	Patient satisfaction, patient experience, telemedicine use, treatment changes	High satisfaction, widely used, preference for continued use
Muller et al. [8]	Randomized Controlled Trial	Non-acute headache disorders	402	Norway, tertiary care	Video consultations	Traditional in-person consultations	NR	Feasibility, acceptability, consultation time, travel cost, diagnostic accuracy	Feasible, comparable Outcomes, reduced time and costs
Seng et al. [13]	Open-label single-arm clinical trial	Migraine and comorbid depressive symptoms	16	USA, urban health system	Telephone and video-delivered MBCT	NR	Pre-post (no control)	Feasibility, acceptability, treatment fidelity, headache disability, depressive symptoms, usability	Feasible, high usability, reduced disability and depressive symptoms
Arena et al. [14]	Pilot feasibility study/case series	Vascular headache (migraine and combined migraine-tension headache)	4	NR	Psychophysiological treatment via telemedicine	None	NR	Feasibility, efficacy	Feasible; preliminary Symptom improvement
Robblee and Starling [24]	Quality improvement pilot study	Headache disorders	75 + additional cohorts	USA, academic headache clinic	E-consultation	Face-to-face consultations	NR	Feasibility, appropriateness, triage, waiting time	Feasible, reduces in-person visits, requires proper triage
Kleiboer et al. [15]	Comparative study	Migraine	44/31 vs. 31	Netherlands	Online digital assistance (ODA)	Behavioral training alone	6 months	Feasibility, acceptability, compliance, migraine outcomes	Feasible, good compliance, no additional clinical benefit
Mueller et al. [16]	Retrospective cohort study	Headache disorders	1390	USA, tertiary care	Remote communications (patient portal, messaging, telephone)	NR	NR	Utilization patterns, demographic factors, clinical factors	Higher use in chronic headache, lower in older patients
Hwang et al. [17]	Retrospective cohort study	Headache disorders	74vs 222	USA, urban academic medical center	eConsult (interprofessional electronic consultation)	In person referral	12 months	Health care utilization, ambulatory visits, referral outcomes	No increase in healthcare utilization vs. in-person referral
Downes et al.[18]	Retrospective matched cohort study	Headache disorders	74 vs. 74	USA, academic medical center	eConsult	Matched in-person referral group	6 months	Healthcare utilization, outpatients visits, emergency department visits	Reduced outpatient visits, no increase in ED use
Andruskevicius et al. [19]	Cross-sectional survey study	Migraine	847	Lithuania, nationwide	Teleconsultations	NR	NR	Telemedicine use, patient experience, treatment changes, patient preferences	Effective, well accepted, preference for hybrid care
Schaetz et al.[20]	Non-interventional cohort study	Migraine	141	Switzerland, corporate setting	Telemedicine consultation+telecoaching" app	NR	6–9 Months	MIDAS, PAM, patient satisfaction, productivity, cost analysis	Improved outcomes and productivity, high satisfaction, cost savings
Muller et al., [10]	Randomized Controlled Trial	Non-acute headache	402	Norway, tertiary care	Telemedicine consultations	3 and 12 months	HIT-6, VAS	Safety	Non-inferior efficacy and safety vs. in-person
Grasso et al. [21]	Prospective observational study	Primary headache	45	Italy, outpatient clinic	Telemedicine follow-up (video consultations)	NR	2 visits (4–8 weeks)	Feasibility, patient satisfaction, usability, communication quality, time/cost savings	Feasible, high satisfaction, improved communication and cost/time savings

**Table 2:** Characteristics of the secondary literature included in this narrative review, including narrative reviews, systematic reviews, meta-analyses, consensus statements, guidelines, and position papers evaluating telemedicine in headache disorders

Author, Year	Study Type	Population	*N* of included studies	Main Findings
Arca et al. [4]	Position statement/Narrative Review	Headache disorders	NR	Non-inferior; improves access/satisfaction; reduces costs; safe
Rogers et al. [22]	Narrative Review	Headache disorders and pain disorders	NR	Improves access; supports multidisciplinary care; expands delivery modalities
Belvis et al. [5]	Consensus- Guidelines	Headache disorders	NR	Increased use post-COVID-19; expanded modalities, recommended for stable follow-up; tailored use
Noutsios et al. [9]	Narrative Review	Patients with non-acute headache	NR	Non-inferior; structured assessment; digital tools, higher satisfaction; limited by exam/technical issues
León-Salas et al. [25]	Systematic Review and meta-analysis	Neurological diseases (including headache)	25 RCTs (n:2335)	Improves outcomes/QoL, function, utilization); strongest evidence in stroke; limited evidence in other neurological conditions
Spina et al. [23]	Narrative Review	Headache/Migraine	32 studies	Feasible; virtual assessment; digital follow-up; tele-rehab potential; limited by tools/exam issues
Bentivegna et al. [26]	Narrative Review	Headache/Migraine	NR	Valid tool; supports diagnosis/monitoring; expanded post-COVID-19; integration potential.

Overall, randomized controlled trials and observational studies have shown that telemedicine-based consultations are associated with clinical outcomes comparable to those obtained with traditional visits [21]. Headache-related disability, pain intensity, and overall symptom burden—assessed using validated instruments such as the Headache Impact Test (HIT-6), the Migraine Disability Assessment (MIDAS), and visual analogue scales (VAS)—generally do not show significant differences between telemedicine-based management and in-person care [9]. These findings consistently support the non-inferiority of telemedicine compared with traditional care in terms of short- and medium-term clinical outcomes in selected populations of patients with headache.

However, the interpretation of non-inferiority results requires caution. Most available randomized studies were conducted in highly selected populations of patients with non-acute headache and within specialized centers, potentially limiting generalizability to broader clinical contexts. Furthermore, follow-up durations were often limited to short- or medium-term outcomes, and long-term effectiveness data remain scarce.

Safety data reported in the studies further reinforces the clinical validity of telemedicine in headache management. No increase in adverse events, missed diagnoses, or clinical worsening has been observed in patients managed remotely compared with those evaluated in person [17, 18]. However, it is important to emphasize that the safety of telemedicine appears to be closely linked to its implementation within well-defined clinical pathways and to the adoption of appropriate patient selection criteria [24].

Evidence from randomized non-inferiority studies provides particularly strong support for the use of telemedicine in headache management, demonstrating durable symptom control and comparable safety profiles over time. Taken together, these data suggest that telemedicine may represent a reliable alternative to conventional consultations for patients with stable headache who require continuous monitoring rather than an in-depth neurological evaluation [22, 25].

Despite these encouraging results, the currently available evidence remains limited. Most studies have been conducted in specialized headache centers and have included carefully selected populations, often excluding patients with acute presentations or warning symptoms. Moreover, the inability to perform a complete neurological examination represents an intrinsic limitation of telemedicine, highlighting the need for hybrid care models that integrate remote and in-person assessments.

Overall, current evidence supports the clinical effectiveness and safety of telemedicine in the management of headache in non-acute and follow-up settings. When integrated into structured care pathways, telemedicine can ensure outcomes comparable to those of traditional visits while maintaining adequate safety standards. However, its role in the evaluation of acute headache and in initial diagnostic assessments remains poorly explored and requires further investigation [15, 26]. A more practical perspective involves identifying which patients are most suitable for telemedicine and which may require in-person evaluation. Telemedicine appears particularly appropriate for follow-up visits, management of patients with established diagnoses, treatment monitoring, medication adjustments, and behavioural interventions. In contrast, first-time evaluations, acute headache presentations, suspected secondary headaches, and complex diagnostic cases generally require in-person neurological assessment. In this context, hybrid care models, integrating telemedicine with traditional consultations, may represent the most effective and safe approach across different clinical scenarios.

## Patient experience, acceptance, and engagement

Across the studies included in this review, the use of telemedicine in headache management was consistently associated with high levels of patient satisfaction. Telemedicine interventions were delivered through heterogeneous modalities, including video consultations, telephone calls, e-mails, messaging systems, and dedicated applications. Despite this variability, patient acceptance was found to be high regardless of the specific technological modality adopted [12].

The reported benefits were mainly related to increased convenience and a reduction in the burden associated with in-person visits. Patients frequently highlighted economic advantages, such as reduced travel costs and fewer work absences, as well as significant time savings resulting from the elimination of travel and waiting times in hospital settings. The possibility of receiving care within the home environment was also perceived as a relevant factor, contributing to higher overall levels of satisfaction and engagement [11, 13, 14, 20, 23].

While patient satisfaction is consistently reported as high, it is important to consider that a substantial proportion of studies were conducted during or shortly after the COVID-19 pandemic, a period characterized by restricted access to in-person care. This contextual factor may have influenced patient perceptions and acceptance of telemedicine, potentially leading to an overestimation of its long-term preference in routine, non-emergency settings. Further longitudinal research is needed to determine whether the high levels of acceptance observed during the pandemic are maintained over time under routine clinical conditions.

However, acceptance of telemedicine was not uniform across all populations. Higher levels of satisfaction and participation were observed particularly among younger patients and those more familiar with digital technologies [16]. Some studies suggest, however, that adequate patient education and repeated use of telemedicine tools during follow-up may promote a progressive improvement in acceptance even among individuals with lower levels of technological literacy [27]. However, the risk of marginalizing segments of the population with limited technological literacy remains significant. Consequently, further in-depth analysis is warranted to better characterize this demographic and ensure equitable access to care.

These findings also raise the issue of potential digital exclusion. Patients with limited digital literacy, inadequate access to technological resources, or lack of caregiver support may be disadvantaged by care models predominantly based on telemedicine, highlighting the importance of inclusive implementation strategies.

Finally, the available evidence consistently indicates that telemedicine is perceived as particularly appropriate for follow-up visits in headache management, whereas its use in first visits remains limited. The inability to perform a complete neurological examination remotely represents a relevant barrier to the use of telemedicine in initial diagnostic assessments and in the management of acute headaches. Not surprisingly, most available studies have focused on follow-up settings, with still limited data supporting the use of telemedicine for first visits or acute presentations [19]. For this reason, telemedicine appears to be an excellent tool for supporting patients throughout their therapeutic journey, whereas its use for the initial patient contact remains significantly limited.

## Real-world implementation and health system impact

From a healthcare system perspective, telemedicine has the potential to significantly improve efficiency and resource utilization in headache management. By reducing the need for in-person visits, telemedicine can decrease waiting times, optimize specialist availability, and lower indirect costs related to travel and work absence. Furthermore, it may enhance access to care, particularly for patients living in underserved or remote areas, and improve continuity of care through more flexible and timely follow-up strategies. These aspects are particularly relevant in chronic conditions such as headache disorders, where repeated consultations are often required. In this context, telemedicine may also contribute to reducing inappropriate emergency department visits and improving care pathway efficiency. Beyond clinical aspects, telemedicine-based care models may also have relevant economic and organizational implications. Available evidence suggests potential reductions in healthcare costs and resource utilization, including decreased emergency department visits, reduced travel-related expenses, and improved efficiency of care delivery. In particular, cost analyses have shown that telemedicine interventions may lead to significant savings at both the patient and system levels [28] while umbrella reviews conducted in OECD countries have highlighted their potential to improve sustainability and efficiency of healthcare services [29] However, further high-quality studies are needed to confirm these findings in headache-specific populations.

The development of telemedicine in the management of headache disorders has occurred rapidly, often in response to sudden and unforeseen clinical needs. specifically, several telemedicine models have been successfully implemented in clinical practice, including structured tele-follow-up programs, remote triage systems, and hybrid care pathways integrating virtual and in-person visits. These approaches have demonstrated the potential to optimize resource allocation and improve continuity of care in patients with headache disorders. However, their large-scale adoption remains dependent on regulatory frameworks, reimbursement policies, and data security standards, which vary across healthcare systems and may represent barriers to implementation [8].

However, for telemedicine to be stably and effectively integrated into routine clinical practice, its implementation must take place within clearly defined regulatory and organizational frameworks established by healthcare systems [30]. Recent studies have further explored the feasibility of web-based behavioral and multimodal therapeutic programs in patients with headache. In particular, pilot experiences have demonstrated that interventions combining patient education, lifestyle modification, and online mindfulness-based therapies may lead to significant improvements in headache frequency, medication use, and patient-reported outcomes, both in adolescent and adult populations [31, 32].

A central aspect concerns the security and protection of sensitive data. The absence of structured regulations may encourage the use of informal communication modalities, such as unprotected emails or messages, which do not meet the necessary requirements in terms of privacy and security. The adoption of dedicated and certified platforms therefore represents a fundamental prerequisite to ensure the protection of health information and the quality of care.

Effective implementation of telemedicine also requires adequate training for both patients and healthcare professionals. Patients should be provided with clear instructions and support tools for the correct use of technologies, while physicians require specific training pathways, considering that a significant proportion of the medical workforce may not be familiar with advanced digital tools. Training therefore represents a key element in reducing barriers to adoption and improving the effectiveness of telemedicine visits [33].

Additional critical issues concern organizational aspects, particularly scheduling, reimbursement, and the integration of telemedicine visits into existing care pathways. The definition of clear criteria for access and reimbursement of telemedicine services could help reduce inappropriate consultations and promote a more structured and secure use of available resources.

Finally, the implementation of telemedicine should consider the risk of excluding populations that are less familiar with technology or lack adequate support from caregivers. Inclusive organizational models that actively involve both patients and healthcare professionals are essential to ensure equity of access and to prevent the widening of healthcare disparities [34].

## Gaps, limitations, and future directions

Despite the growing interest in telemedicine, the available evidence remains limited. In particular, there are still few randomized controlled studies that have systematically investigated the use of telemedicine, both in general neurological settings and, more specifically, in the management of headache disorders. In most cases, the available evidence derives from studies conducted in specialized centers, involving highly selected populations, thereby limiting the generalizability of the findings. Moreover, substantial heterogeneity exists across studies in terms of telemedicine modalities (video consultations, telephone-based care, asynchronous eConsult systems, and digital therapeutic platforms), patient selection criteria, and outcome measures. This variability limits direct comparability and makes it difficult to define standardized models of care. The absence of unified implementation frameworks and consensus-based appropriateness criteria further highlights the need for structured clinical pathways.

Another important gap concerns the near-total lack of studies evaluating the effectiveness and safety of telemedicine in emergency or urgent care settings. Emergency department access for headache represents one of the most frequent reasons for neurological consultation, and the use of telemedicine in such contexts could potentially improve access to specialist care, particularly in centers without on-site neurologists, while simultaneously reducing management costs and the need for patient transfers.

However, the inability to perform a complete neurological examination remotely represents a relevant limitation to the application of telemedicine in acute settings. At the same time, the development of structured protocols for remote assessment could allow neurologists to identify cases requiring in-person diagnostic evaluation or higher levels of care, while maintaining safety and clinical appropriateness.

Overall, future research should focus on prospective, randomized studies including more heterogeneous populations and diverse clinical settings, in order to more precisely define the role of telemedicine throughout the entire headache care pathway. The integration of hybrid care models and standardized protocols represents a promising direction to overcome current limitations.

Telemedicine, however, should not be considered as an exclusive tool nor as a complete substitute for in-person visits. On the contrary, its use appears to be more effective when embedded within integrated care models, in which in-person and remote assessments complement each other according to the clinical context and patient characteristics.

For telemedicine to be adopted sustainably, it is essential that both patients and healthcare professionals become familiar with these tools and that their use is supported by appropriate training pathways. The progressive integration of telemedicine into clinical practice therefore represents a first step toward the development of increasingly specific and structured protocols, in which access modalities, appropriateness criteria, and reimbursement systems are clearly defined [4, 5].

In this scenario, telemedicine can significantly contribute to the evolution of care models in headache management, provided that it is implemented in a regulated, inclusive manner and centered on patient needs.

In addition to the limitations of the current literature, some limitations of this review should be acknowledged. First, this is a narrative review and does not follow a formal systematic review methodology, which may introduce selection bias. Second, the literature search was primarily conducted using PubMed, potentially excluding relevant studies indexed in other databases. Third, no formal quality assessment of included studies was performed. Finally, the included evidence is heterogeneous and comprises both primary studies and secondary sources, which may limit the comparability and generalizability of the findings.

## Conclusions

Telemedicine, particularly in the follow-up of chronic and non-acute headache conditions, represents a valuable tool that can be effectively integrated into routine clinical practice. Current evidence suggests that, in appropriately selected patients, telemedicine ensures clinical effectiveness and safety comparable to traditional in-person visits, while improving continuity of care and access to specialist services.

However, telemedicine should not be considered a complete substitute for face-to-face evaluation. Its greatest potential emerges when incorporated into integrated care pathways, where remote and in-person consultations are combined according to clinical context and patient characteristics.

In this context, telemedicine may also facilitate the implementation of structured therapeutic programs, particularly non-pharmacological interventions such as behavioral therapies, patient education, and self-management strategies. These approaches may further enhance patient engagement and contribute to long-term disease management.

For sustainable implementation, structured organizational models, appropriate training for healthcare professionals and patients, and clear regulatory frameworks are essential. Future research should prioritize the evaluation of telemedicine in acute and emergency headache settings, where current evidence remains limited. In addition, further studies are needed to define optimal models of care that integrate telemedicine with in-person evaluations. Hybrid care pathways may represent the most effective strategy to ensure both accessibility and clinical safety across different stages of headache management.

In this perspective, telemedicine may contribute meaningfully to the evolution of headache care, provided that it remains patient-centered, inclusive, and clinically appropriate [20].
